# Oblique-view video tracking and density-based counting: accurate counting of late-stage rapeseed seedlings for breeding assessment

**DOI:** 10.3389/fpls.2026.1770912

**Published:** 2026-02-17

**Authors:** Bowen Luo, Yuang Yang, Kuanyan Zhang, Xuan Lv, Yujie Liu, Yicheng Yang, Fugui Zhang, Lu Liu, Gang Zhang, Xiaole Wang, Zhenchao Wu

**Affiliations:** 1 School of Engineering, Anhui Agricultural University, Hefei, Anhui, China; 2 School of Agriculture, Anhui Agricultural University, Hefei, Anhui, China; 3 Anhui Agricultural University New Countryside Development Research Institute Wandong Comprehensive Test Station, Chuzhou, Anhui, China; 4 Anhui Provincial Engineering Research Center for Intelligent Agricultural Machinery Equipment, Hefei, Anhui, China; 5 School of Intelligent Manufacturing, Anhui Science and Technology University, Fengyang, Anhui, China

**Keywords:** adaptive DBSCAN, field-based phenotyping, rapeseed seedlings counting, tracking, YOLO

## Abstract

Accurate counting of late-stage rapeseed seedlings is critical for yield estimation and field management, while traditional manual counting is inefficient and labor-intensive, calling for an automated counting method. A novel video tracking and counting method (CropTriangulator) was proposed, which uses smartphone-captured videos to achieve row-based accurate counting based on oblique view and target density distribution. It integrates three core components: YOLOv11n was selected for its balanced detection accuracy and inference speed after model comparison; an adaptive DBSCAN (AdapDBSCAN) algorithm was designed to eliminate non-target seedlings by dynamically adjusting parameters to address perspective distortion; the SORT algorithm was adopted for tracking and counting, with permanent ID marking to ensure uniqueness when seedlings cross frame boundaries. Experiments on 20 test videos (10 for 45° oblique view, 10 for 90° vertical view) showed that CropTriangulator achieved an average counting accuracy of 97.13% at 45° (14% higher than 90°), with the R-squared of 45° row-based counts reaching 0.917. AdapDBSCAN reduced over-counting compared with fixed-parameter DBSCAN, and SORT had a much lower ID switch rate (8.47%) than DeepSORT (36.05%). The 45° oblique view is proven optimal for rapeseed seedling counting. The proposed CropTriangulator provides a low-cost and efficient solution for automated row-based counting in complex field environments, supporting precise yield estimation and scientific field management decisions. The video comparing the effects of the CropTriangulator method is available at: https://github.com/Possibility007/Comparison-of-counting-results.git

## 1 Introduction

Seedling emergence rate is one of the main bases for rapeseed breeding and field management, which traditionally relies on time-consuming and labor-intensive manual field observation and counting, thus highlighting the urgent need for automated methods. Breeding superior varieties has become a high-priority demand in the agricultural market, and achieving uniform seedling emergence is key to crop breeding (Paparella et al., 2015; Sun et al., 2023). As a critical indicator of crop phenotypic traits, seedling emergence rate is usually calculated by counting the number of germinated seeds and the total number of sown seeds. Traditionally, these data are obtained through manual counting, which is inefficient and costly (Zhuang et al., 2024). By acquiring images of rapeseed seedlings and processing them using deep learning techniques, automatic target detection and quantity statistics of rapeseed seedlings can be realized, providing strong support for seed selection, breeding, and optimization of field management measures.

Deep learning techniques have been widely applied to automate target detection and quantity statistics, showing potential in identifying rapeseed seedlings in complex scenarios from images (Zhao et al., 2018). To address the effective identification of different parts of tomato plants, Cardellicchio et al. (2023) proposed a model based on YOLOv5 single-stage detectors (both standalone and ensemble detectors) to automatically identify and extract key phenotypic traits from tomato images under various stress conditions. Rong et al. (2019) applied two different convolutional neural network structures to walnut images to solve the problem of rapid detection of foreign objects in walnuts, achieving an accuracy of 99.5% in automatically segmenting images and detecting natural foreign objects of different sizes. Khaki et al. (2022) proposed a new deep learning framework, WheatNet, which can accurately and efficiently count wheat ears in the field, collecting real-time data for farmers to make scientific and reasonable wheat planting and management decisions. Misra et al. (2020) proposed an object detection-based method to automatically identify and count wheat ears from images. Additionally, a wheat ear counting method based on frequency domain decomposition was proposed (Bao et al., 2023), significantly improving the accuracy of wheat ear counting in images to 91.5%. For single images, deep learning techniques perform well in crop target detection and counting, but they cannot cover all objects in a single field.

The identification of rapeseed seedlings in a single image usually fails to reflect the total number of targets in a single field, which can be addressed using video tracking. To solve the low efficiency of traditional manual monitoring of peanut seedling emergence rate in fields, Lin et al. (2022) proposed a real-time peanut video counting model (combining improved YOLOv5s and DeepSort), achieving a counting ability close to that of humans with an accuracy of 98.08%. Tan et al. (2022) improved the cotton seedling tracking method by combining a one-stage target detection deep neural network with optical flow, providing an automatic and near-real-time video tracking method that achieves high-precision seedling detection under high occlusion, image blur, complex backgrounds, and extreme lighting conditions, with an average precision of 99.12%. Tan et al. (2023) developed a plant seedling and flower counting method using an anchor-free deep convolutional neural network-based tracking approach, conducting experiments on 75 cotton seedling videos and 50 cotton flower videos collected in fields, with average relative errors of 5.5% and 10.8%, respectively. Rong et al. (2023) proposed an improved tomato cluster counting method combining target detection, multi-object tracking, and counting in specific tracking regions, addressing the challenges of automated tomato yield estimation in practical applications and realizing tomato cluster yield estimation in greenhouse scenarios with an accuracy of 97.9%. Barreto et al. (2021) successfully achieved fully automated counting of sugar beets, maize, and strawberries by combining a UAV-based camera system with deep learning algorithms, with errors below 4.6%. A major advantage of crop detection using image data is the ease of implementing application algorithms, while using video data for crop monitoring is more conducive to practical field applications (Lin et al., 2022). For field crops, video data are usually acquired from a top-down overhead view, which struggles to accurately capture crop features in complex scenarios.

Video tracking from an overhead view typically achieves good results in the early stages of crop growth. However, during rapeseed breeding, different varieties exhibit varying growth statuses: even when sown simultaneously, some varieties (e.g., mustard-type rapeseed) remain in the early seedling stage, while others (e.g., cabbage-type rapeseed) may enter the late seedling stage, where stems and leaves overlap as they grow, posing challenges for rapeseed counting. Chen et al. (2024) proposed a regression deep learning-based visual model, HOB-CNNv2, to segment tree branches under extreme occlusion using data acquired from the side of fruit trees. Chen et al. (2023) proposed a lightweight multi-class occluded target detection method for Camellia oleifera fruits, testing data acquired from different oblique angles and improving detection accuracy loss caused by multiple occlusion types, with an average precision of 94.1%. Zheng et al. (2022) optimized YOLOv4 to address the impact of leaf occlusion on tomato detection accuracy for picking robots, achieving an average detection accuracy of 94.44% when the camera angle is 90° relative to the ground. From an overhead view, most bottom leaves are covered or obscured, hindering comprehensive analysis of the entire crop. From an oblique view, the entire plant structure is visible, enabling quantification of leaves, plant height, and branch area (Zhang et al., 2019). Therefore, data acquired from different oblique views is expected to distinguish individual rapeseed plants for accurate counting of rapeseed seedlings.

Even though rapeseed plants can be distinguished from an oblique view, due to linear perspective, crop morphology in videos acquired from oblique angles usually exhibits distortion (smaller in the distance and larger in the foreground), affecting clustering accuracy and thus counting accuracy. To address perspective issues, Dolata et al. (2021) proposed an adaptive nonlinear regression model that adaptively adjusts parameters to match the morphological characteristics of different plants, predicting the contour of each plant in online-acquired images with an accuracy of 86.9%. Liu A. et al. (2025) proposed a threshing gap adaptive adjustment system based on feed rate monitoring and established a feed rate monitoring model, with an average precision of 90.8% for the system. Zhang et al. (2023) corrected distorted images from oblique views by adaptively adjusting perspective transformation, solving the vanishing point problem commonly occurring at the top of parallel crop rows. Adaptively adjusting the clustering radius is expected to address minor distortion in videos caused by linear perspective, enabling accurate counting of crops in fields.

In summary, the aforementioned studies on static multi-view data acquisition and adaptive adjustment methods provide various insights for target counting. However, existing methods still have room for optimization in counting at the late seedling stage. In particular, rapeseed seedlings exhibit complex growth states in actual field scenarios, requiring counting methods to balance efficiency and accuracy. Therefore, integrating current research results with practical application needs, this study proposes a video tracking and counting method for rapeseed seedlings at the late seedling stage based on oblique view and target density distribution, using easily operable smartphones to acquire videos of late-stage rapeseed seedlings, realizing accurate counting of rapeseed seedlings in target regions. Firstly, YOLOv11 was utilized to detect rapeseed seedlings from 45° oblique and 90° vertical views. Secondly, rapeseed seedlings in target regions were extracted based on target density distribution. Then, extracted rapeseed seedlings were assigned IDs and counted. Finally, target detection and counting performance from the two views are compared, and the superior view was selected for the counting method in this study.

## 2 Materials and methods

**Figure 1:**
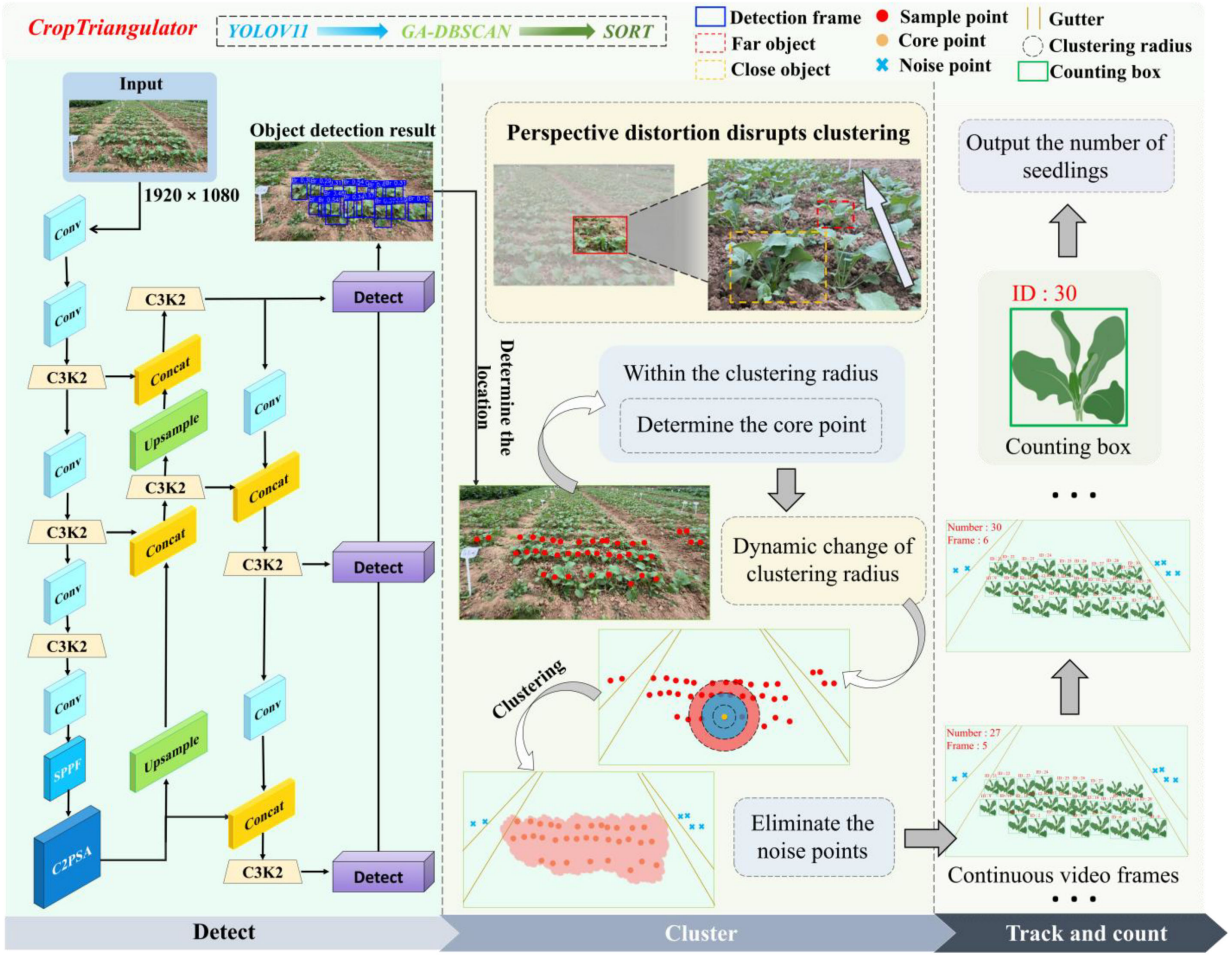
Workflow of the method, including detection, clustering, tracking, and counting.

An automatic rapeseed seedling counting method for calculating the seedling emergence rate in modern rapeseed fields is proposed in this study. The method workflow is shown in **Figure 1**. Firstly, videos of rapeseed seedlings at different oblique angles were collected in modern rapeseed fields to construct a dataset. Secondly, different scales of YOLOv11 models were trained and compared to achieve accurate counting of rapeseed seedlings. Thirdly, based on YOLOv11n detection of rapeseed seedlings in videos, the AdapDBSCAN algorithm was proposed to eliminate rapeseed seedlings in non-target regions. Fourthly, after eliminating rapeseed seedlings using the AdapDBSCAN algorithm, the SORT algorithm was utilized to assign IDs to the extracted rapeseed seedlings and count them. Finally, a rapeseed seedling video counting method, CropTriangulator, was proposed, integrating three core modules: YOLOv11 target detection, adaptive DBSCAN clustering, and SORT counting algorithm, forming a complete computer vision processing pipeline. Detailed information outlining each step is provided below.

### 2.1 Dataset

#### 2.1.1 Study area

**Figure 2:**
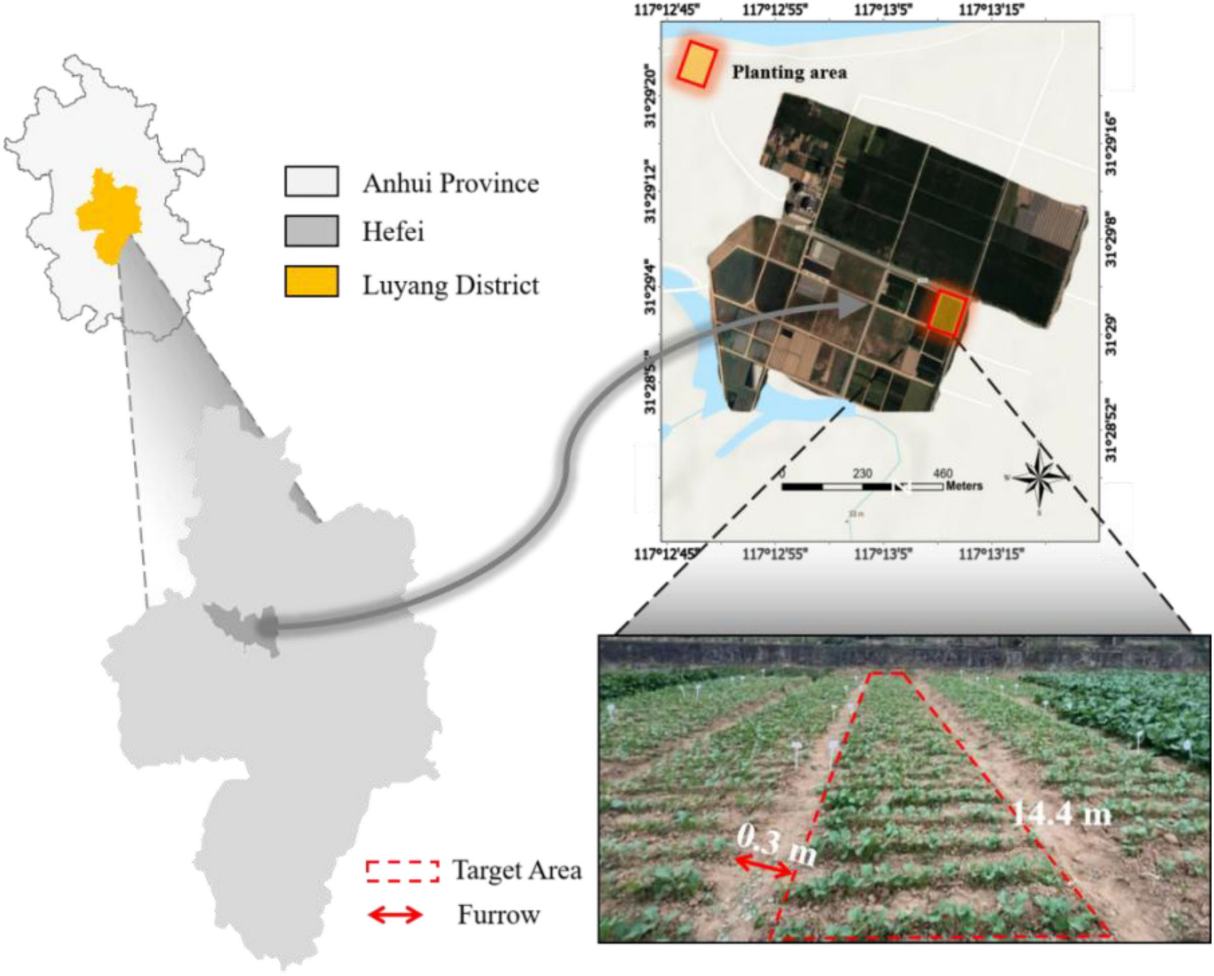
Data collection area. The red solid line area represents the rapeseed planting area, the red dashed line area represents a complete data acquisition area, and the red arrow area represents the width of the drainage ditch.

The study area is located at the Teaching and Demonstration Base of Anhui Agricultural University in Hefei, Anhui Province, China (N: 31°29′4.36″, E: 117°13′23.97″, altitude 47 m), belonging to the northern subtropical humid monsoon climate zone, as shown in **Figure 2**. A total of 108 rapeseed varieties, including Brassica rapa, Brassica juncea, and Brassica napus, were selected. The spacing between each rapeseed plant is 0.05 m, and furrows are dug on both sides of the planting area for irrigation and waterlogging drainage (i.e., row spacing of 0.3 m). Each variety is planted in plots (1.2 m in width and 14.4 m in length).

#### 2.1.2 Data acquisition

**Figure 3:**
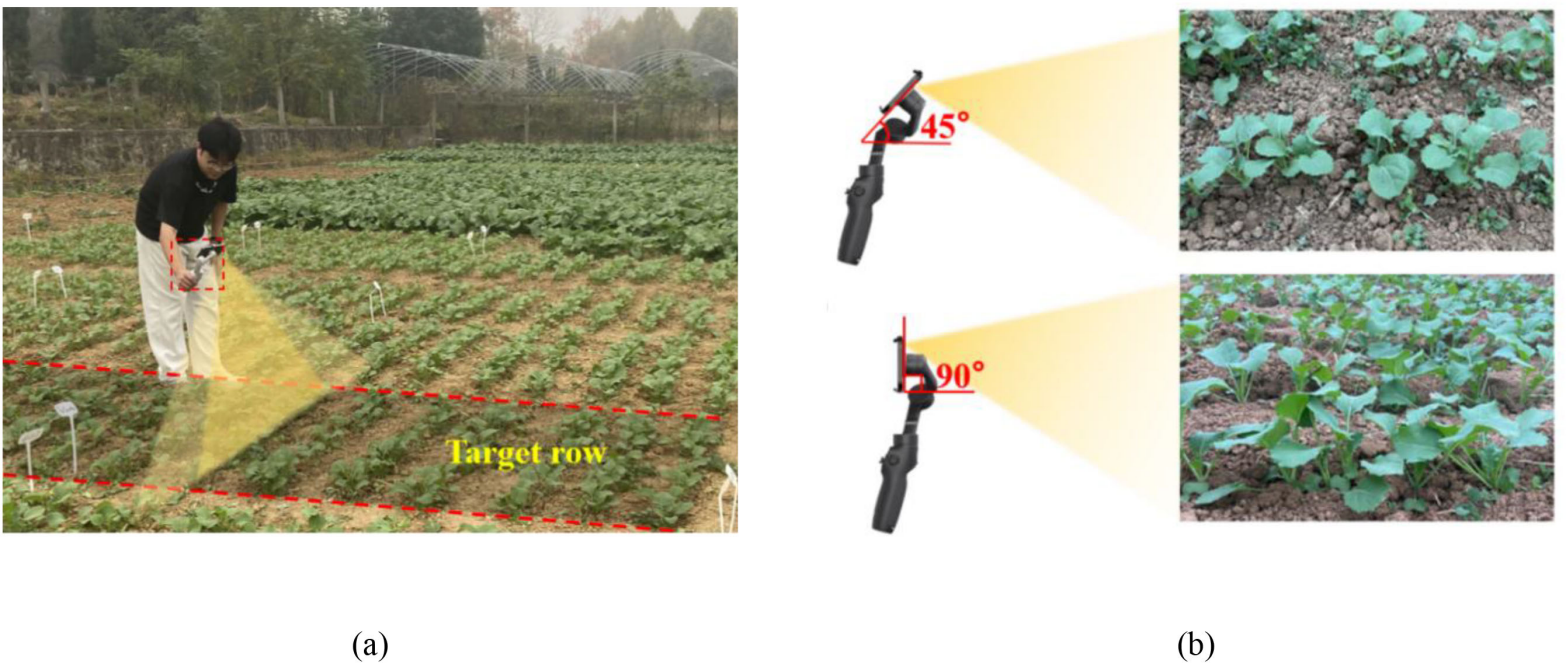
**(a)** Staff holding a smartphone fixed on a DJI gimbal walking along the furrow to acquire data, where the light yellow area represents the maximum horizontal angle range that can be captured, and the yellow area represents the target row captured by the camera; **(b)** example images of rapeseed plants under 45° oblique view and 90° vertical view set by the gimbal.

The boundary of the rapeseed seedling population between two adjacent furrows is defined as a row unit, from the beginning to the end within the planting area. Staff held an iPhone 14 Pro Max (Apple Inc., California, USA) fixed on a DJI Osmo Mobile SE gimbal (DJI, Shenzhen, China), walking at a constant speed along the furrow from the beginning of each plot to acquire complete video data of each row of rapeseed seedlings (image resolution: 1920 × 1080 pixels, frame rate: 30 frames per second, number of videos: 28), with the tester’s walking speed of approximately 0.8 m/s, as shown in **Figure 3a**. During data collection, the weather was initially clear before transitioning to partly cloudy conditions. The iPhone 14 Pro Max camera was adjusted to ultra-wide-angle mode, and the DJI Osmo Mobile SE gimbal was set to pitch-lock mode, with video shooting angles divided into a 45° angle (referred to as the 45° oblique view, abbreviated as 45° view) and a 90° angle (referred to as the 90° vertical view, abbreviated as 90° view) between the phone and the ground, as shown in **Figure 3b**, at a height of approximately 0.5 m from the ground. After data collection, the staff manually count the number of rape seedlings in each row and record it as the actual number of rape seedlings in each row, which was called ground truth (*GT*) and was utilized to analyze and discuss the tracking and counting performance.

#### 2.1.3 Dataset preparation and data processing

**Figure 4:**
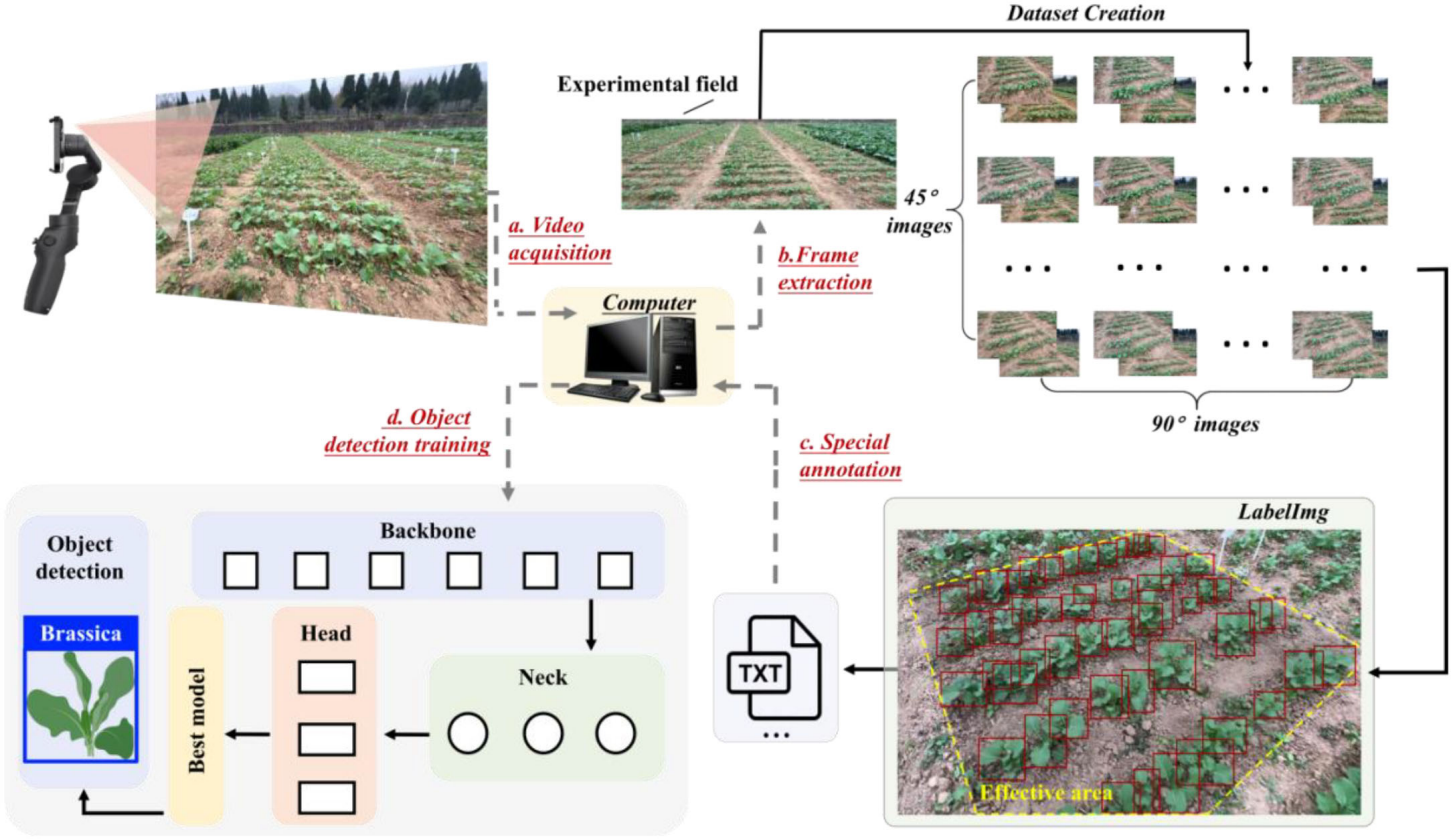
Data processing workflow. **(A)** Data acquisition; **(B)** dataset creation; **(C)** special annotation strategy, where the area framed by the yellow dashed line represents the region meeting the annotation strategy, the area outside the frame represents the non-annotated region, and the red rectangular box represents the annotation box from LabelImg; **(D)** dataset training.

From 28 collected videos on November 15, 2024 (14 for 45° view and 14 for 90° view), 8 videos (4 per view) were selected, with each video having a fixed frame rate of 30 fps, duration of 20 seconds, and original resolution of 1920×1080 pixels. A sampling strategy of extracting 1 frame from every 6 consecutive frames (5-frame interval) was adopted, which generated 800 images (400 per view). This approach avoids redundant overlapping frames (which would reduce diversity if the sampling interval were reduced), while ensuring the richness of the data, covering the complete video sequence, 108 rapeseed varieties with diverse phenotypes, and complex field conditions. The remaining 20 videos were employed to validate the performance of the rapeseed counting model. The dataset was randomly divided into training, validation, and test sets in an 8:1:1 ratio for training the detection model. Detailed dataset information is shown in **Table 1**. Secondly, the image dataset was manually annotated using LabelImg, following the method of Liu D. et al. (2025). It is worth noting that a special strategy was adopted for dataset annotation: only valid regions within target rows of images were annotated, as shown in **Figure 4**(iii). The area formed by annotated rapeseed seedlings in an image is referred to as the valid region under the special annotation strategy. For rows where rapeseed seedlings can be clearly displayed in full, all seedlings were annotated; for rows where more than half of the rapeseed seedlings are blurred or incompletely displayed, the entire row was not annotated. The total annotation time was approximately 200 hours. Rectangular bounding boxes indicate the positions of seedlings. After manual annotation, TXT files containing target types and coordinate information were generated for training on the dataset. The total data processing workflow is shown in **Figure 4**.

**Table 1:** The detailed information of data acquisition.

Collection date	Collection views	Number of videos	Initial image resolution	Number of images	Train dataset	Test dataset	Acquisition time
Nov 15, 2024	45° view	14	1920×1080	400	320	80	8:00~13:00
Nov 15, 2024	90° view	14	1920×1080	400	320	80	13:00~17:00

Rapeseed seedling detection provides a basis for counting. Rapeseed seedling detection faces unique challenges due to its classification as dense object detection. As a representative of one-stage target detection algorithms, YOLOv11 performs excellently in small-scale dense target detection, combining high precision and speed (Huang et al., 2025). The performance of different scales of YOLOv11 models was compared and analyzed, and the optimal model was selected for rapeseed seedling detection.

In this study, network training was implemented on a desktop computer equipped with an Intel Core i9-12900K (3.19 GHz) CPU, NVIDIA GeForce RTX 3090 GPU, 16 GB RAM, and 64-bit Windows 10. Specific experimental configurations are shown in **Table 2**. The training batch size was 16, epochs were 500, and image size was 960 (Daniels et al., 2021). The original resolution of the images in the dataset is 1920×1080 pixels. Due to the large number of pixels in the original images, the model requires excessive computational resources during training, resulting in slow training speed. Moreover, a resolution of 960 pixels is sufficient to retain the features of crop seedlings without causing accuracy loss due to scaling. To meet the computational efficiency and input size requirements of YOLOv11 model training, a long-side scaling strategy is adopted during training to uniformly adjust the images to a long-side length of 960 pixels. Five models with different performances of YOLOv11 were trained on datasets from 45° and 90° views, respectively. Then, the models with the highest precision (*AP*) and optimal inference time were selected for video tracking from 45° and 90° views, respectively. All models were trained on the constructed dataset with the same predefined parameters to ensure consistency and comparability.

**Table 2:** The specifications details of hardware and software.

Configuration	Parameter
CPU	Intel Core i9–12900 K(3.19GHz)
GPU	NVIDIA GeForce RTX 3090
Operating system	Windows 10
Accelerated environment	CUDA12.6 CUDNN8.9.7
Development environment	Pycharm 2023

### 2.2 Rapeseed seedling density distribution clustering method based on perspective adaptive adjustment (AdapDBSCAN)

**Figure 5:**
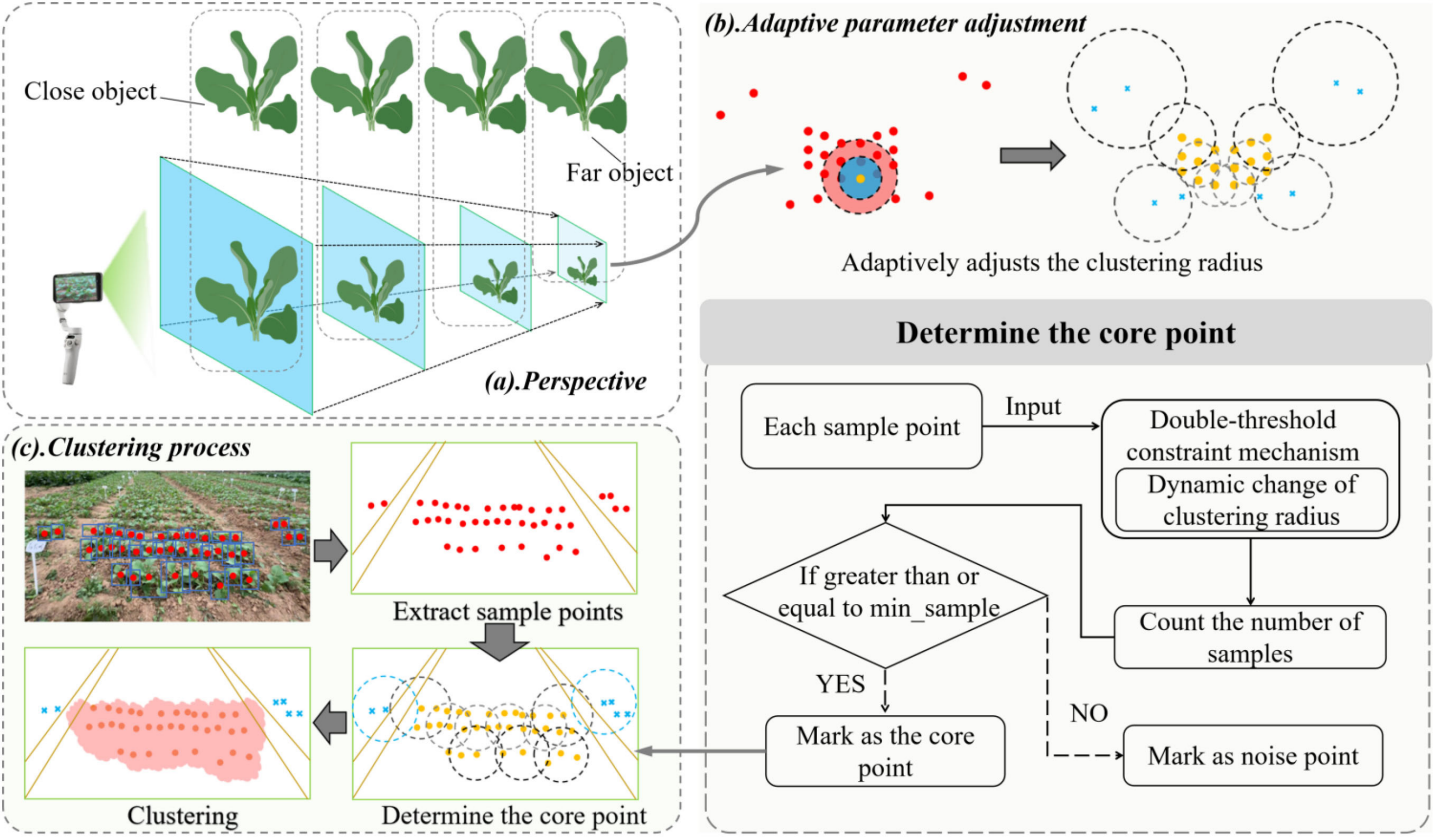
**(A)** Perspective-induced “small in distance and large in foreground” of rapeseed seedlings; **(B)** adaptively adjusting clustering radius; **(C)** workflow of the AdapDBSCAN algorithm. The area surrounded by brown lines represents the drainage ditch, red points represent samples, yellow points represent core points, blue crosses represent noise points, and dashed circles of different colors represent different clustering radius.

Due to linear perspective effects, the acquired video data in this study exhibits distortion, with rapeseed seedling density appearing denser in the distance and sparser in the foreground along the shooting direction, and the pixel values occupied by rapeseed seedlings showing smaller in the distance and larger in the foreground, as shown in **Figure 5a**.

DBSCAN is a classic density-based unsupervised clustering algorithm that enables data-driven clustering via neighborhood density thresholds (i.e., clustering radius eps and minimum sample count min_samples) (Ester et al., 1996; Cheng et al., 2024). However, the two core parameters of the original DBSCAN algorithm are fixed and necessitate manual tuning, which renders it inadequate to accommodate the “small in distance and large in foreground” perspective distortion of rapeseed seedlings in oblique-view videos, resulting in erroneous clustering in areas with adjacent density variations. Existing adaptive DBSCAN variants (e.g., Khan et al., 2018) are predominantly optimized for general-purpose scenarios and do not account for the unique density distortion characteristics of crops under field oblique viewing conditions. To address the perspective-induced “small in distance and large in foreground” challenge, an adaptive DBSCAN parameter-tuning method (AdapDBSCAN) is proposed. A parameter optimization method based on local density was introduced in this study, constructing a nonlinear mapping relationship between the eps parameter and regional target density, while establishing a dynamic calculation model for the min_samples parameter. The center point of the detection box is taken as the position of each detected rapeseed seedling, with each center point treated as a sample point representing a rapeseed seedling. Firstly, a nonlinear functional relationship between the eps parameter and local regional target density is constructed to dynamically adjust the eps: when the number of samples in a region is dense, the eps value decreases; when the number of samples is sparse, eps increases. Secondly, the number of samples within the eps is counted to dynamically calculate the min_samples parameter: when the number of samples in a region is denser, the number of core point min_samples is larger; when the number of samples is sparser, the number of core point min_samples is smaller. Finally, based on this functional relationship, starting from any point *x*, if the number of samples within the eps of point *x* is greater than or equal to min_samples, point *x* is marked as a core point; otherwise, it is marked as a noise point. Core points form clusters, and noise points are marked as candidate elimination set *N* and eliminated during clustering, as shown in **Figure 5b**. Through dynamic determination of parameters twice, rapeseed seedlings in non-target regions (i.e., sparse seedlings detected on both sides) are eliminated. Specific formulas are shown in Equations 1, 2. The process is shown in **Figure 5c**, with the red area representing the target region.

(1) $$eps=\max(1,\frac{\Delta v}{\alpha })\times median(D_{filtered})$$

(2) $$\text{min}\_samples(k)=[\frac{\sum_{x=1}^{k}\rho_{x}}{k\cdot \delta }]$$

where *Δv* is the vertical span, *α* is a normalization constant utilized to map the vertical span *Δv* to a scaling factor; *median(D_filtered_)* is the filtered horizontal spacing of rapeseed seedlings (i.e., filtering out outliers such as excessively large spacing) to avoid interference of extreme values on the reference distance. *ρ_x_* is the neighborhood density of the *x* sample within the eps, and *δ* is the density decay factor.

In addition, rapeseed seedlings in non-target regions have small imaging sizes and sparse distribution in images. If not controlled, DBSCAN is forced to continuously increase eps until covering the entire image to meet the min_sample threshold, leading to clustering failure (all points are classified into the same cluster or noise). In this study, the image size is 1920×1080 pixels. Considering that metric coordinates in images are depth-dependent, a global pixel-to-actual-distance mapping was not adopted. Instead, based on camera calibration at a fixed shooting height (0.5 m) and actual measurement of the target region, the average pixel-to-distance ratio in the central area of the image (where target seedlings are concentrated) was approximately 800 pixels per meter. When the maximum threshold of eps exceeds the width of the drainage ditch, a broader non-target area enters. Here, the spacing between the rapeseed seedlings has further increased. Even if the eps is further expanded, it is difficult to gather min_sample samples within the neighborhood. Eventually, it falls into a cycle of infinitely increasing the eps to meet the threshold. Based on this, according to the image spatial scale and the actual width of the drainage ditch (0.3 meters), the maximum threshold of eps was preset to 250 pixels. The corresponding actual space is approximately 0.3125 meters, which has exceeded the width of the drainage ditch. And a dual-threshold collaborative constraint mechanism was constructed with the filtered horizontal spacing of rapeseed seedlings (*median(D_filtered_)*) to effectively suppress unlimited expansion of the eps parameter.

### 2.3 Rapeseed seedling counting method: CropTriangulator

Representative algorithms were compared via controlled experiments. The SORT algorithm was ultimately selected.

Integrating previous research, the rapeseed seedling counting method integrated YOLOv11, AdapDBSCAN, and SORT to realize the counting of rapeseed seedlings in target regions. To better count rapeseed seedlings, when the geometric center of a counting box (for counting seedlings) crosses the lower edge of the video frame, the counting box is automatically removed, and its unique identifier (ID) assigned by the SORT algorithm is permanently marked to ensure no reuse in subsequent frame processing, thus ensuring ID uniqueness (Wu et al., 2023b). This counting method eliminates non-target rows through the AdapDBSCAN algorithm, only counts target regions, and displays the current number of detected rapeseed seedlings and frame number in the upper left corner of the video.

### 2.4 Application development of CropTriangulator

**Figure 6:**
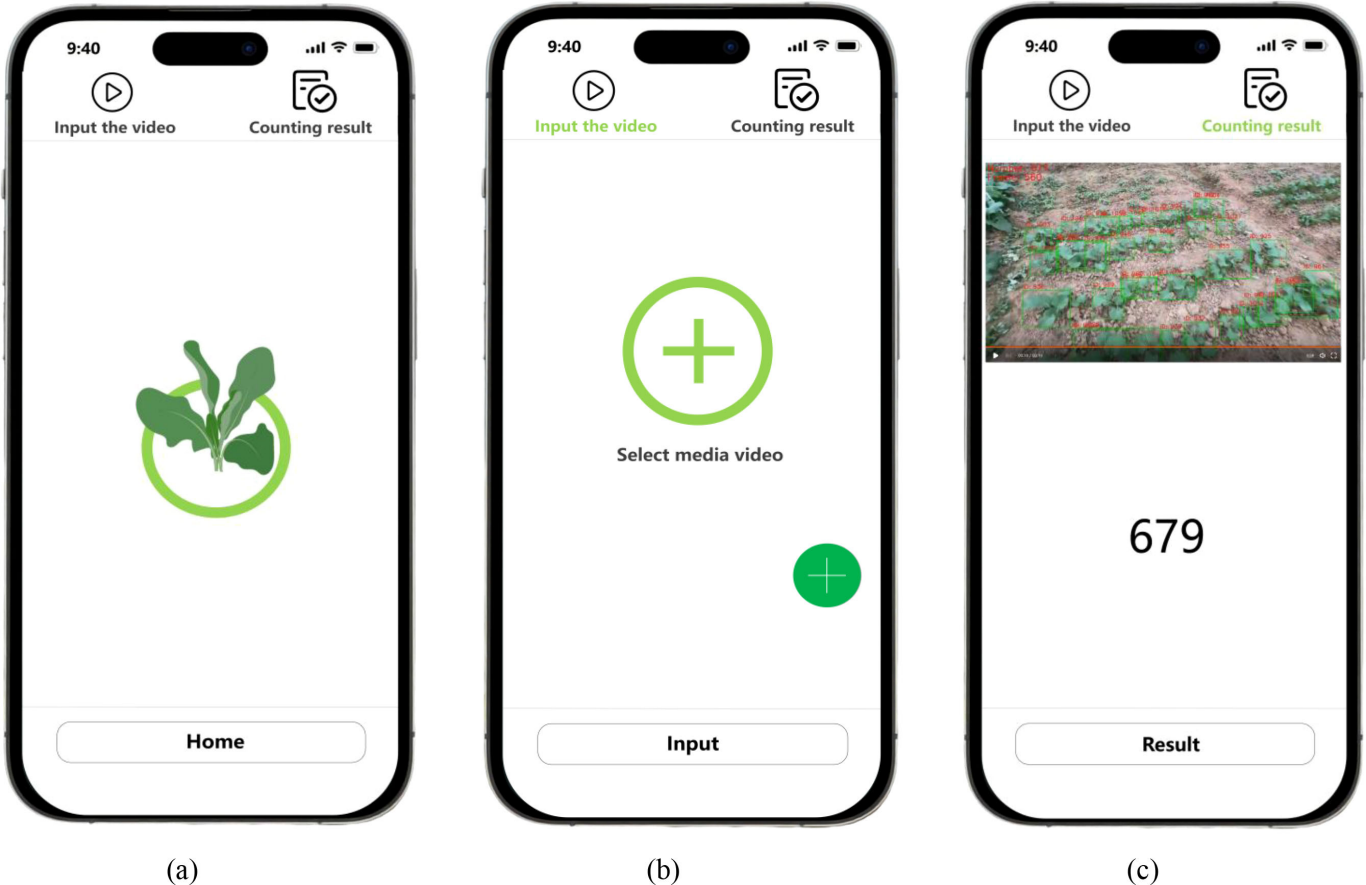
The interfaces of the client application. **(a)** The home interface of the client application. **(b)** The functional interface of the client application. **(c)** The output interface for row-based rapeseed seedling counting results.

The mobile client implementation of the CropTriangulator system adopts a three-tier architectural framework comprising: (1) a user interface (UI) layer built with native UI components for video upload and visualization; (2) an interaction layer managing file selection and processing requests through native event handlers; and (3) a presentation layer generating dynamic result displays. The system incorporates mobile-optimized UI design with screen-adaptive layouts, file picker functionality providing visual feedback, and animated processing indicators. The user operation interfaces are illustrated in **Figure 6**.

The operational workflow begins when users upload field-captured videos through the functional interface. These video files are transmitted to the backend server via HTTP protocol, where they are queued for processing. The server executes the complete CropTriangulator analysis pipeline, including YOLOv11-based seedling detection, AdapDBSCAN clustering for target region identification, and SORT algorithm for seedling tracking and counting. Upon completion, the processed results are returned to the client application. Users can view the rapeseed seedling count calculated by the CropTriangulator method on the counting results interface, as shown in **Figure 6c**. The entire client application is implemented with native mobile development technologies to ensure platform compatibility and maintainability.

### 2.5 Evaluation indicators

#### 2.5.1 Target detection performance indicators

In this study, to correctly evaluate the accuracy of target detection, commonly used indicators in the deep learning field were adopted: precision (*P*) and recall (*R*), with calculation formulas shown in Equations 3, 4. Average precision (*AP*), an indicator reflecting the performance of target detection models (Yang J. et al., 2025; Qin et al., 2025), is defined by *P* and *R*, as shown in Equation 5.

(3) $$P=\frac{TP}{TP+FP}$$

(4) $$R=\frac{TP}{TP+FN}$$

(5) $$AP=\sum \left(P_{\text{i}}\cdot \text{Δ}R_{\text{i}}\right)$$

where *TP* (True Positives) is the number of correctly predicted positive instances; *FP* (False Positives) is the number of instances predicted as positive but actually negative; *FN* (False Negatives) is the number of positive instances not identified by the model; *AP* is the average precision.

#### 2.5.2 Tracking performance and counting performance indicators

Three commonly used indicators in the tracking field were utilized to evaluate the performance of rapeseed tracking in videos: ID switch rate (*W_ID_*), target tracking accuracy (*P_tr_*), and target tracking precision (*P_mt_*) (Wu et al., 2023a). The calculation formulas for these indicators are shown in Equations 6-8.

(6) $$W_{ID}=\frac{Q_{\text{sh}}}{S}$$

(7) $$P_{\text{tr}}=\frac{M_{\text{mat}}}{S}$$

(8) $$P_{\text{mt}}=\frac{M_{\text{mat}}}{T_{\text{mat}}}$$

where *Q_sh_* is the number of rapeseed seedlings with ID switches during counting in the experimental video; *S* is the number of rapeseed seedlings counted by the SORT algorithm in the experimental video, with each *S* corresponding to a uniquely determined video; *M_mat_* and *T_mat_* are the number of correctly tracked rapeseed seedlings and the total number of matched rapeseed seedlings in the experimental video, respectively.

The performance of the rapeseed seedling counting model was evaluated using the accuracy (*Acc*) metric, as defined in Equation 9, which is utilized to evaluate the performance of the CropTriangulator method and different oblique angles. This indicator measures the ratio of correctly counted rapeseed seedlings to the total number of rapeseed seedlings in the row across 20 test videos from 45° oblique and 90° vertical views. According to the shooting order and angle of the videos, the test videos are denoted as Video *i* (*i* = 1, 2,…, 20), where Videos 1 to 10 represent videos shot from 45° view, and Videos 11 to 20 represent videos shot from 90° view. Videos 1 to 10 and Videos 11 to 20 form pairwise counterparts, each pair capturing the same row from different views.

(9) $$A\text{cc}=(1-\frac{\left|\hat{y}-y\right|}{y})\times 100\%$$

where *y* represents the actual number of rapeseed seedlings in the target row of the video (*GT*), and $\hat{y}$ represents the number of rapeseed seedlings counted by the algorithm. The R-square (*R*^2^), which is defined by Equation 10, is employed to assess the overall error between the counting of rapeseed seedlings and *GT*.


(10) $$R^{2}=1-\frac{\sum_{\text{i}=1}^{10}{\left(y_{i}-\hat{y}\right)}^{2}}{\sum_{\text{i}=1}^{10}{\left(y_{i}-\bar{y}\right)}^{2}}$$

where *y_i_* represents the number of rapeseed seedlings in Video *i*, and $\bar{y}$ represents the average number of rapeseed seedlings per row at 45° or 90° shooting angles.

## 3 Result and discussion

### 3.1 Performance of object detection model

**Figure 7:**
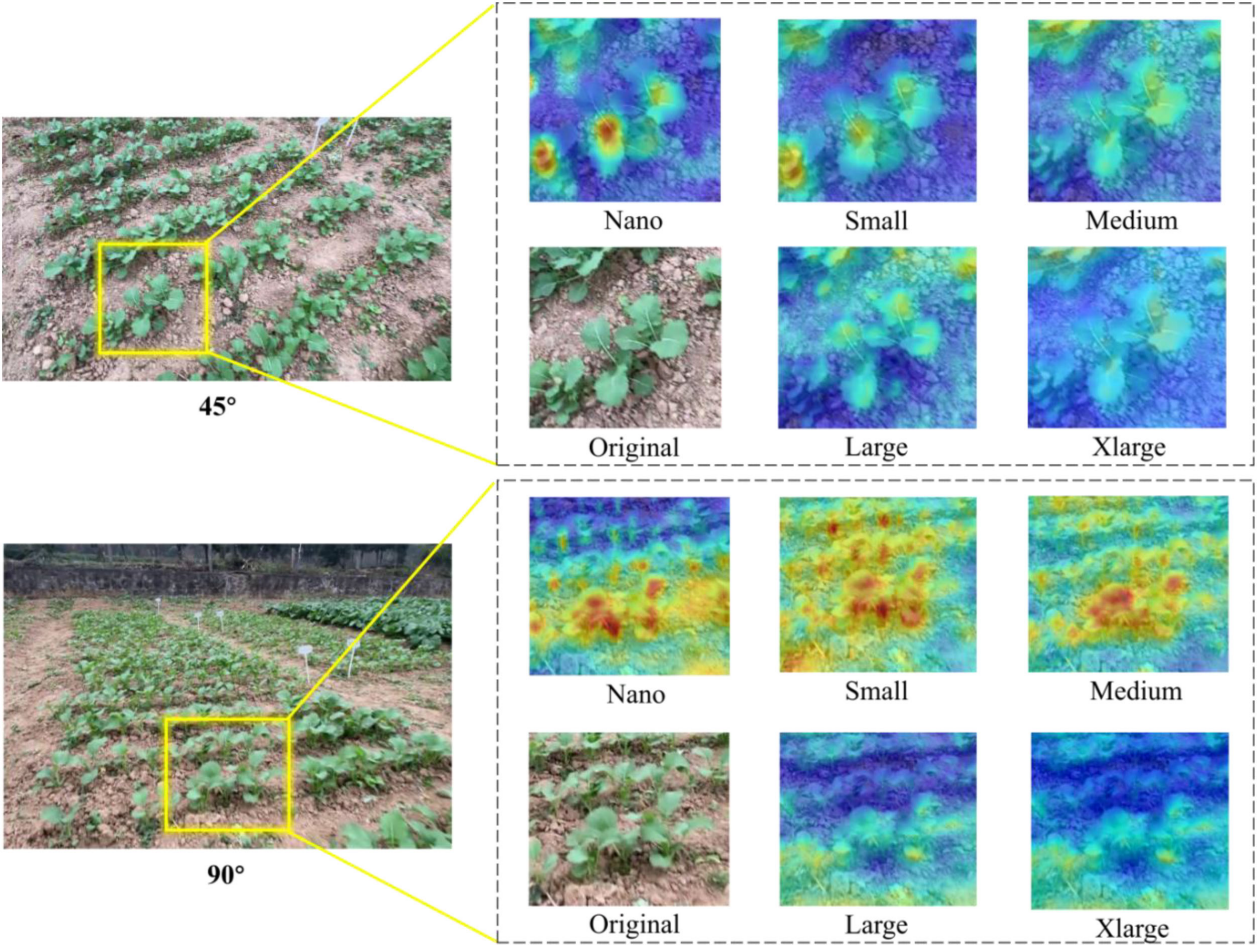
Heatmaps from different angles. Yellow boxes represent randomly selected comparison regions, and red regions represent areas with more attention in the model.

Different scales of YOLOv11 models all performed well in rapeseed seedling detection, as they balanced *AP* and inference time. The *AP* and inference time of different scales of YOLOv11 models from different angles are summarized in **Table 3**, with the best scores highlighted in bold. As the number of parameters in the feature extraction network increased, *AP*_0.5_ and *AP*_0.5:0.95_ for both 45° and 90° views showed a downward trend, and inference time also increased. The nano-scale and medium-scale models for the 45° view had the same *AP*_0.5_ and *AP*_0.5:0.95_ values, but the medium-scale model had an inference time 115% slower than the nano-scale model, while the nano-scale model achieved the fastest inference time and required the smallest model size. For the 90° view, the nano-scale model had the highest *AP*_0.5_ and *AP*_0.5:0.95_ and the shortest inference time. **Figure 7** shows visualization heatmaps generated by Grad-CAM (Yang Y. et al., 2025), intuitively presenting the regions focused on by target detection models of different scales. The brightness of each region indicates its importance to the model, with higher brightness representing stronger attention. Results showed that the nano-scale model performed best for the 45° view, and the small-scale model performed best for the 90° view, but with little difference from the nano-scale model. YOLOv11n outperforms larger-scale YOLOv11 models due to its superior adaptation to late-stage rapeseed seedling detection scenarios, rather than being constrained by dataset scale. Late-stage rapeseed seedlings exhibit relatively uniform morphological and spectral features compared to general targets, leading larger models with excessive parameters to overfit trivial background noise (e.g., soil texture variations, isolated weed pixels) in field images. In contrast, the streamlined architecture of YOLOv11n (incorporating Focus and C3k2 modules) prioritizes the extraction of core seedling features (e.g., leaf contours, stem-root connections), enabling it to achieve the same *AP*_0.5_ and *AP*_0.5:0.95_ values as the medium-scale model. By comprehensively considering detection accuracy, computational efficiency, and heatmap results, the nano-scale model has advantages in detecting small-scale targets, so it was selected for target detection in both 45° and 90° views.

**Table 3:** Results of YOLOv11 models in different scales.

Model scale	*P*		*R*		*AP*_0.5_		*AP*_0.5:0.95_		Inference time/ms	
	45°	90°	45°	90°	45°	90°	45°	90°	45°	90°
Nano	**0.908**	**0.775**	0.850	**0.802**	**0.942**	**0.856**	**0.811**	**0.488**	**4.6**	**3.9**
Small	0.887	0.774	0.876	0.819	0.941	0.850	0.805	0.474	6.0	5.4
Medium	0.869	0.764	**0.887**	0.796	**0.942**	0.826	**0.811**	0.461	9.9	3.3
Large	0.892	0.751	0.865	0.748	0.940	0.777	0.807	0.440	13.4	12.4
Xlarge	0.855	0.728	0.832	0.701	0.932	0.751	0.792	0.415	23.6	19.8

The optimal values for each model under different conditions were displayed in bold.

**Figure 8:**
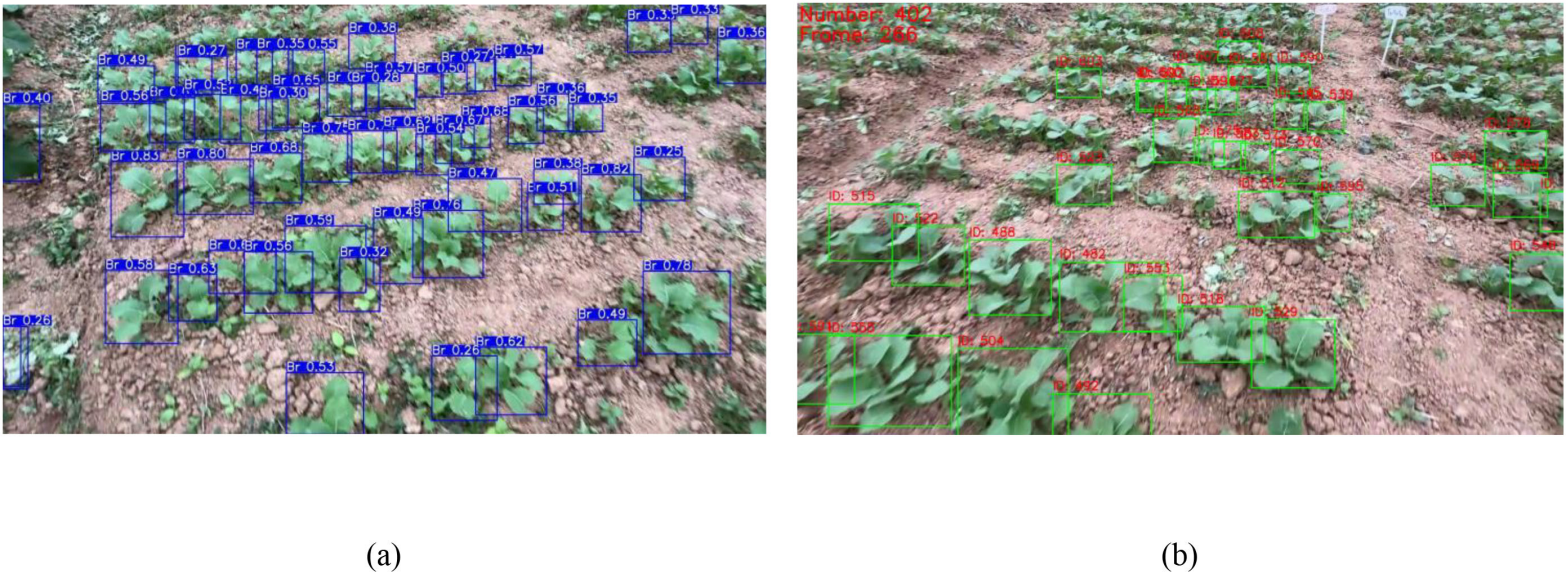
**(A)** Rapeseed seedlings in non-target regions detected; **(B)** impact of detected rapeseed seedlings in non-target regions on counting.

However, the superior performance of YOLOv11 is affected to some extent, leading to detection boxes in unannotated regions. For example, when the model scans complex scenes, its strong feature extraction capability captures elements in unannotated regions that are similar to target features, as shown in **Figure 8a**. This not only generates false detection results in unmarked regions but also complicates subsequent result screening, as shown in **Figure 8b**. Additionally, YOLOv11’s excellent noise reduction and detail enhancement capabilities may parse the fuzzy pixel clusters in unannotated regions into recognizable target forms, causing detection boxes in non-target regions, which interfere with the detection accuracy of the model.

Although some studies have improved model detection performance using multi-source data such as depth information and infrared imaging (Stumpe et al., 2025; Yang Y. et al., 2025), most current smartphones (especially mid-to-low-end models accounting for the majority of the market) do not have dedicated hardware depth sensors (e.g., ToF, LiDAR). While depth can be inferred from stereo cameras based on multi-view geometry, this method is constrained by environmental interference, high computational complexity, and poor adaptability to dynamic field shooting. By focusing on smartphone RGB cameras, convenient data collection has ultimately been achieved for users; as more users upload RGB video data to the cloud, the dataset can be continuously enriched to boost model generalization. To ensure the universality, real-time performance, and scalability of the method, the counting pipeline has been optimized based solely on RGB video. Therefore, with the continuous improvement of future phone performance, smartphones are expected to break through existing hardware limitations and become terminal devices capable of directly acquiring, processing multi-source data, and outputting results.

Among different scales of YOLOv11 models, target detection results from the 45° view were significantly better than those from the 90° view, with *P*, *AP*_0.5_, and *AP*_0.5:0.95_ for the 45° view significantly higher than those for the 90° view. In particular, for *AP*_0.5:0.95_, the selected nano-scale model for the 45° view improved by more than 30% compared to the 90° view, with little difference in response time, providing guidance for tracking and counting performance from different views. As shown in **Table 3**, the precision of the nano target detection model selected for the two oblique views in rapeseed scenarios was 90.8% and 77.5%, respectively, with recall rates of 85% and 80.2%. High precision and stable recall rates provide a basis for reducing target loss during tracking and counting, avoiding overestimation of counting results due to misjudgment of targets.

### 3.2 Performance of rapeseed tracking and counting method

**Figure 9:**
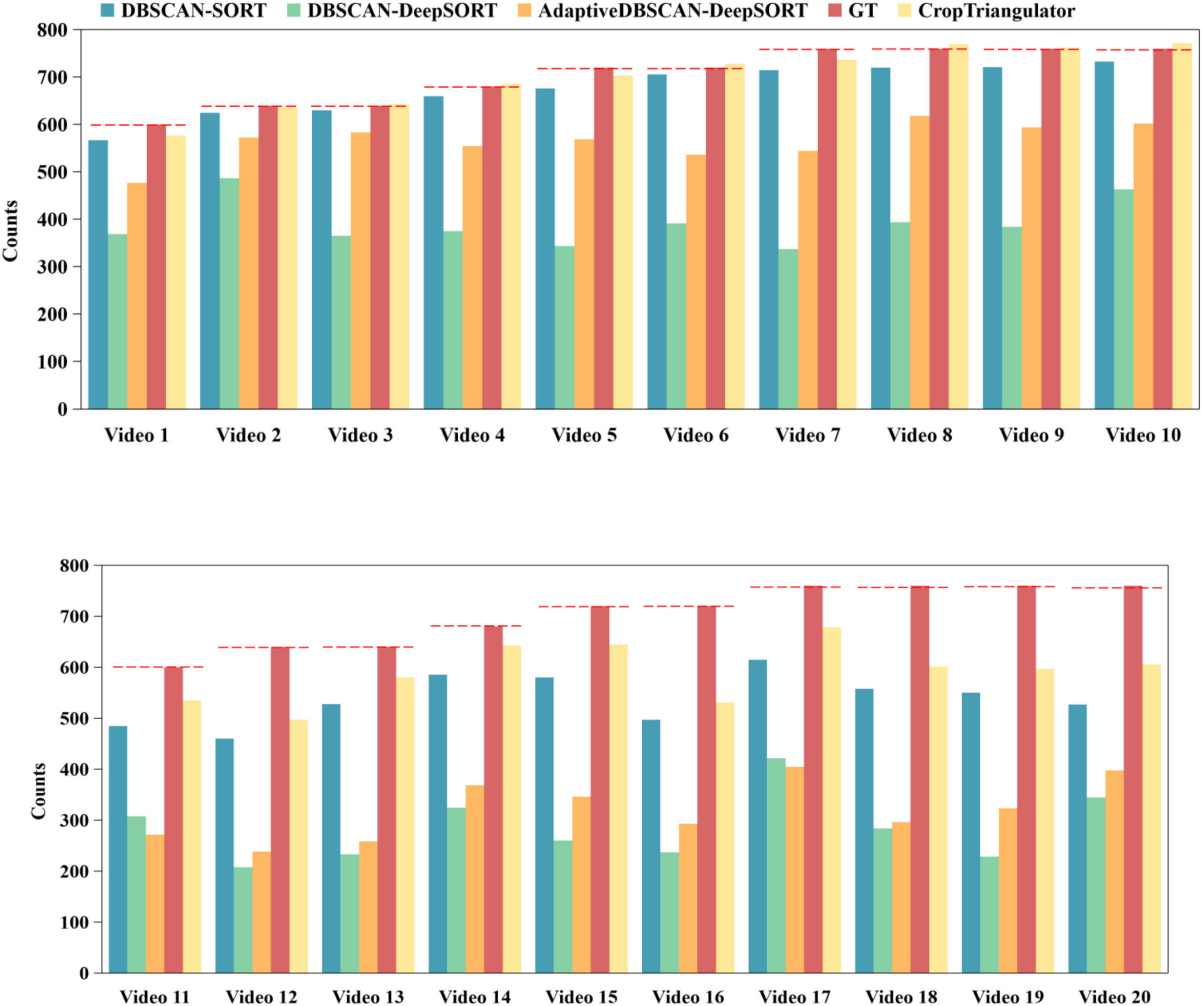
Counting results using different methods. The red dashed line represents the *GT* value of the video. Videos 1 to 10 represent videos shot in order from a 45° view, and Videos 11 to 20 represent videos shot in order from a 90° view. The red dashed line represents the *GT* of the video.

**Figure 10:**
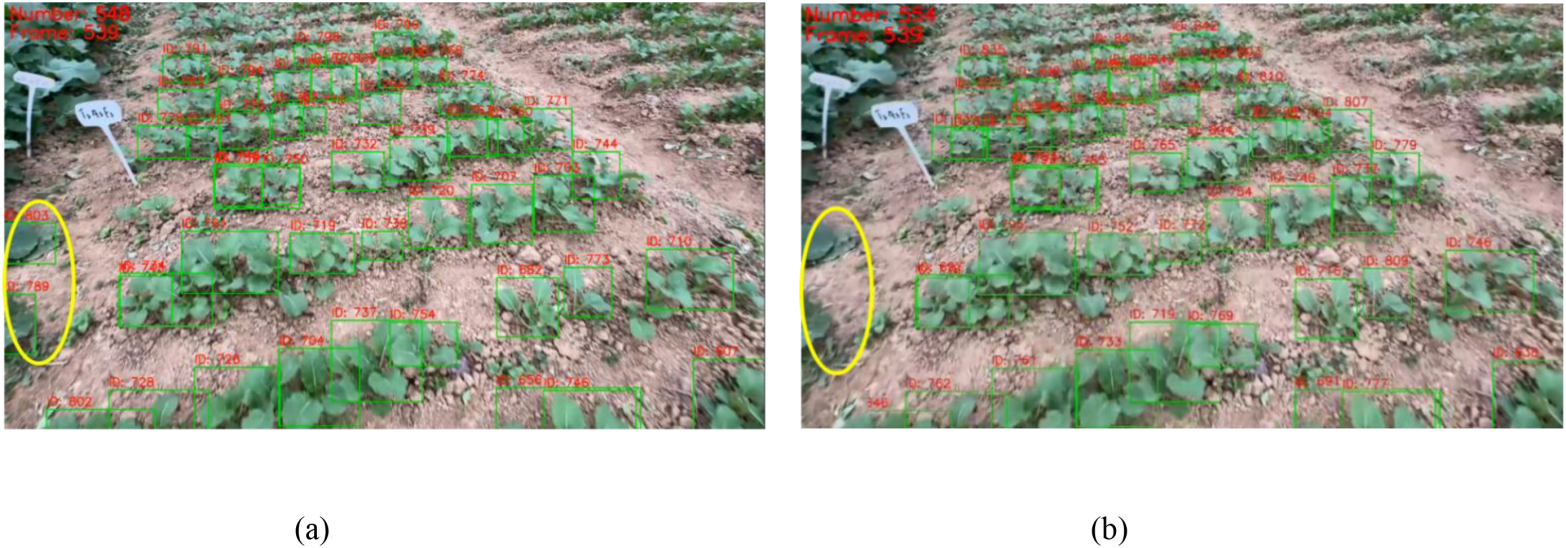
**(A)** Counting result of the 539th frame of Video 4 using the DBSCAN-SORT method; **(B)** counting result of the 539th frame of Video 4 using the CropTriangulator method. Yellow circles indicate regions where detected rapeseed seedlings in non-target rows were effectively filtered out.

The AdapDBSCAN clustering method (adaptive method) based on SORT and DeepSORT exhibited excellent counting performance in processing rapeseed seedling videos captured by smartphones. In addition, the counting method using fixed-parameter DBSCAN clustering based on SORT and DeepSORT (conventional method) showed considerable drawbacks. As shown in **Figure 9**, counting results using the adaptive method for Videos 1 to 20 were closer to the *GT* values, while the conventional method had larger deviations compared to the adaptive method. Taking Video 4 (*GT* = 680) as an example, the counting result using the CropTriangulator method was 679, with an accuracy of 99.85%, and the result using the AdapDBSCAN-DeepSORT method was 554, with an accuracy of 81.47%. Compared to the DBSCAN-SORT and DBSCAN-DeepSORT methods, the accuracy increased by 3.53% and 26.32%, respectively. A comparison of randomly selected frames from Video 4 using the DBSCAN-SORT method and the CropTriangulator method is shown in **Figure 10**. The adaptive method effectively filtered out detected rapeseed seedlings in non-target rows, improving counting accuracy. These results demonstrate the excellent performance and feasibility of the adaptive method.

In a certain frame of the video, due to the geometric characteristics of perspective projection, the actual spatial distribution of distant rapeseed seedlings is compressed in the image, which is manifested as an increase in the number of seedlings per unit image area (i.e., visually increasing density); at the same time, distant rapeseed seedlings occupy fewer pixels in the image, reflecting the impact of projection scaling on target imaging size (Wang Z. et al., 2022). In contrast, due to the short distance from the camera, foreground rapeseed seedlings are less affected by perspective projection scaling, showing sparse spatial distribution in the image, fewer seedlings per unit image area, and more pixels occupied by individual seedlings. This imaging feature of “denser in distance and sparser in foreground” leads to uneven density distribution of acquired rapeseed seedling data (Islam et al., 2024). The conventional method cannot adapt to local density differences, resulting in incorrect clustering and failure to filter out detection results from unmarked regions in the target detection stage, which are instead classified as target row rapeseed seedlings, leading to lower accuracy of the conventional method compared to the adaptive method.

The proposed adaptive method (AdapDBSCAN) fully leverages local target density information and a dual-threshold constraint mechanism to address these inherent limitations: it constructs a nonlinear mapping between the clustering radius (eps) and regional seedling density, dynamically shrinking eps in dense distant regions to maintain precise clustering and expanding eps in sparse foreground regions to preserve valid seedlings, while synchronously adjusting the min_samples parameter based on local sample counts. This scenario-specific optimization enables the adaptive method to accurately identify and separate target rows from non-target areas (e.g., drainage ditches and adjacent rows) even under perspective-induced density variations, effectively filtering out misdetected seedlings in unmarked regions that the conventional method fails to exclude. This approach significantly reduces erroneous clustering associated with fixed parameters, enhances the precision of non-target seedling elimination on both sides of the target rows, and ultimately achieves higher counting accuracy and stability compared to traditional approaches, fully demonstrating its superiority in handling the uneven density distribution of rapeseed seedlings in oblique-view videos.

Perspective distortion introduces three key challenges in practical applications: in the 45° view, the foreground (0-2m from the camera) has a seedling density of 2–3 plants per m² (sparse), while the background (8-14m) reaches 8–10 plants per m² (dense), and fixed-parameter clustering (e.g., traditional DBSCAN) either over-clusters dense regions (merging adjacent seedlings) or under-clusters sparse regions (classifying valid seedlings as noise), leading to ±15% counting error; distant seedlings (background) occupy 3–8 pixels, while foreground seedlings occupy 30–50 pixels, and YOLOv11n’s detection confidence for small targets (less than 10 pixels) drops by 35%, increasing *FN*; the transition zone between target rows and drainage ditches (depth 12-14m) suffers from blurred edges due to perspective compression, leading to 10-12% of *FP* from misclassifying ditch edges as seedlings.

By applying AdapDBSCAN for algorithm optimization, these problems can be solved. The dual-threshold constraint (eps_max=250 pixels and *median_(_D_filtered)_*) dynamically adjusts clustering parameters based on local density. For dense background regions, eps is reduced to 50–80 pixels to avoid over-clustering, and for sparse foreground regions, eps is increased to 150–200 pixels to retain valid seedlings which reduces perspective-induced error by 42% compared to fixed-parameter DBSCAN.

**Figure 11:**
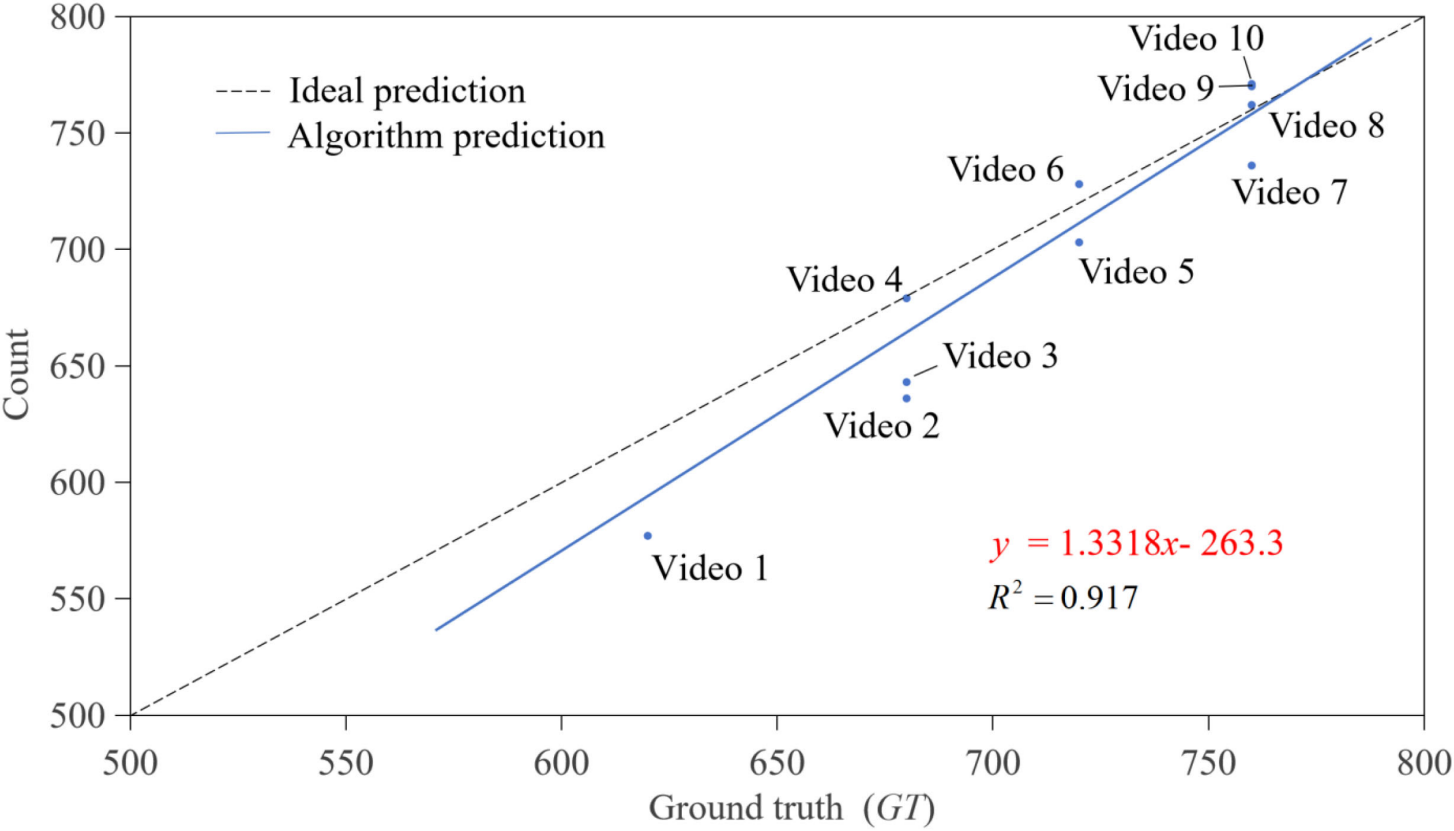
The result of 45° oblique view rapeseed seedling counts calculated based on the CropTriangulator method. The blue scatter represents the counts based on the CropTriangulator method, while the blue solid line represents its corresponding linear regression. The black dashed line represents the ideal counting result.

Furthermore, to compare counting accuracy from different oblique angles, this study systematically evaluated performance differences between 45° oblique view and 90° vertical view. Given the significant advantages of target detection from the 45° view over the 90° view in performance indicators, it is reasonable to infer that superior performance is also expected in target tracking and final result output. Detection from the 45° view can more accurately capture target features, reducing error accumulation in key links such as feature matching and motion prediction, ultimately improving overall tracking performance. Experimental results are shown in **Figure 9**, indicating that for the 45° view, the average accuracy of the DBSCAN-SORT method (94.44%) was approximately 19% higher than that for the 90° view (75.59%); the average accuracy of the CropTriangulator method (97.13%) was more than 14% higher than that for the 90° view (82.94%). Additionally, as shown in **Figure 9**, for the 45° oblique view using the CropTriangulator method, 5 out of 10 videos had an accuracy of over 98%, with the highest at 99.85%, closely matching manual counting results and far exceeding the counting accuracy of the 90° vertical view using the CropTriangulator method. Moreover, linear regression analysis was applied between counting results obtained using the CropTriangulator method and *GT*. As shown in **Figure 11**, the *R*^2^ of counting results from the 45° oblique view was 0.917. These results highlight the excellent counting performance of the 45° oblique view and its strong stability, fully verifying that the 45° oblique view is superior for counting tasks.

Differences in counting performance between angles may stem from three key factors: firstly, the overlap rate of seedlings increases from the vertical view, with foreground seedlings occluding background seedlings; secondly, perspective distortion is enhanced from the 90° vertical view, increasing distortion; finally, rapeseed seedlings from the 45° oblique view contain both root and leaf features, enabling more efficient handling of complex scene interference and accurate counting by the SORT algorithm, while the 90° vertical view contains highly similar crop root features, causing false detections that severely affect target detection and counting performance (Kumar et al., 2014; Liu et al., 2024).

Therefore, future research should focus on constructing more complex datasets containing more detailed and variable video acquisition views. In the future, we will adopt an image distortion correction model to correct the position of each pixel in the image through geometric transformation, restoring spatial relationships between pixels and improving data density consistency (Li et al., 2019). Additionally, efforts should be made to improve the efficiency and flexibility of counting methods, especially in environments with uneven density distribution.

Having established the superiority of the adaptive method and the 45° viewing angle for the framework, the subsequent step involved selecting the most suitable tracking component. For this purpose, control experiments were conducted on various tracking algorithms under the same experimental conditions. Experimental results indicated that BotTrack achieved an average *W_ID_* of 42.56% and a counting accuracy of 59.70% for the 45° view, while ByteTrack exhibited a *W_ID_* of 28.35% and an accuracy of 80.25% for the same view. Both algorithms showed higher *W_ID_* and lower counting accuracy compared to SORT. The *W_ID_* of ByteTrack, although lower than that of DeepSORT, remained substantially higher than the benchmark of 8.47% set by SORT, as shown in **Table 4**. The *W_ID_* (8.47%) of SORT was 19.88% lower than that of ByteTrack and 34.10% lower than that of BotTrack. Furthermore, its counting accuracy (97.13%) was 16.88% and 37.43% higher than those of ByteTrack and BotTrack, respectively.

**Table 4:** Performance comparison of tracking algorithms based on adaptive methods.

Algorithms	*W_ID_* (%)	*P_tr_* (%)	*P_mt_* (%)	45° View *Acc* (%)	Inference time(ms/frame)
SORT	8.47	87.53	89.75	97.13	4.6
DeepSORT	36.05	71.50	54.72	81.47	12.8
BotTrack	42.56	61.30	47.88	59.70	12.4
ByteTrack	28.35	78.62	73.90	80.25	8.7

The inferior tracking stability of BotTrack and ByteTrack in this study can be attributed to two scenario-specific factors. First, despite its plant-targeted design, the feature matching module in BotTrack struggles to distinguish late-stage rapeseed seedlings with highly uniform morphology, leading to frequent ID misassignments and the highest *W_ID_* (42.56%) among all tested algorithms. Its *P_tr_* (61.30%) and *P_mt_* (47.88%) are also the lowest, reflecting poor target association and matching performance under high-similarity conditions. Second, the strategy employed by ByteTrack for associating low-confidence detections, while beneficial in general crowded scenes, becomes counterproductive here. Since the preprocessing stage has already filtered out most noise, the remaining low-confidence boxes largely correspond to ambiguous patches or background clutter. Attempting to associate them introduces unnecessary computational overhead and increases the risk of ID switches, as reflected in its elevated *W_ID_* (28.35%). Moreover, their inference speeds (BotTrack: 12.4 ms per frame; ByteTrack: 8.7 ms per frame) remain significantly slower than that of SORT (4.6 ms per frame), a critical disadvantage for real-time processing in practical deployment.

To focus on balancing efficiency and stability in practical applications and to avoid increasing computational burden and the risk of ID confusion due to the introduction of overly complex feature matching mechanisms, the subsequent comparison of tracking algorithms in this study was concentrated on SORT and DeepSORT. Although algorithms such as BotTrack and ByteTrack excel in general object tracking, their complex feature matching mechanisms were primarily designed for targets with significant appearance differences or variable motion patterns. Under the specific conditions where seedling features were nearly identical and motion was solely induced by camera movement, these sophisticated mechanisms not only struggled to provide effective discrimination but also could increase the risk of ID switches and computational overhead by relying on easily confusable appearance features. This approach aimed to more clearly demonstrate the advantages of a streamlined and efficient tracking framework (SORT) over deep trackers dependent on appearance features (DeepSORT) in the high-similarity scenario of rapeseed seedlings.

**Figure 12:**
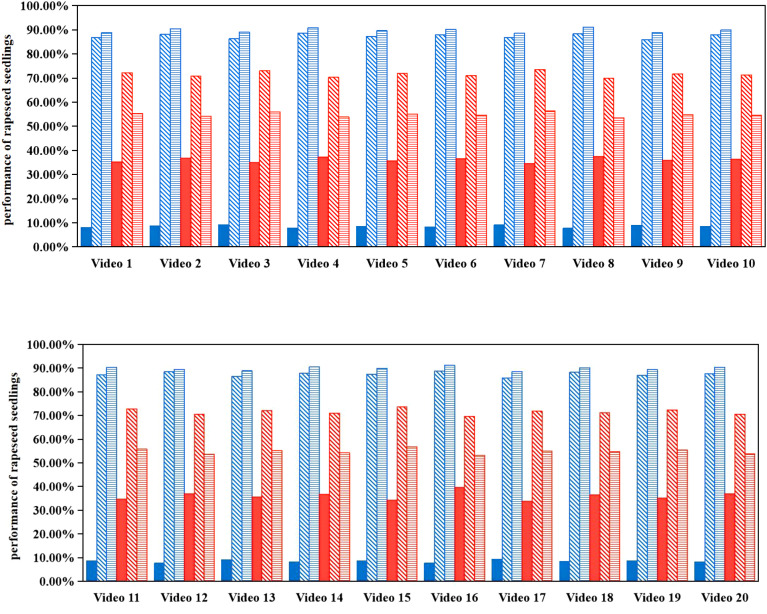
Tracking results of rapeseed seedlings using adaptive counting methods based on SORT and DeepSORT. Results of the adaptive counting method based on SORT are marked in blue; results of the adaptive counting method based on DeepSORT are marked in red; colored filled bars represent ID switch rate (*W_ID_*) results; diagonal bars represent target tracking accuracy (*P_tr_*) results; horizontal bars represent target tracking precision (*P_mt_*) results.

**Figure 13:**
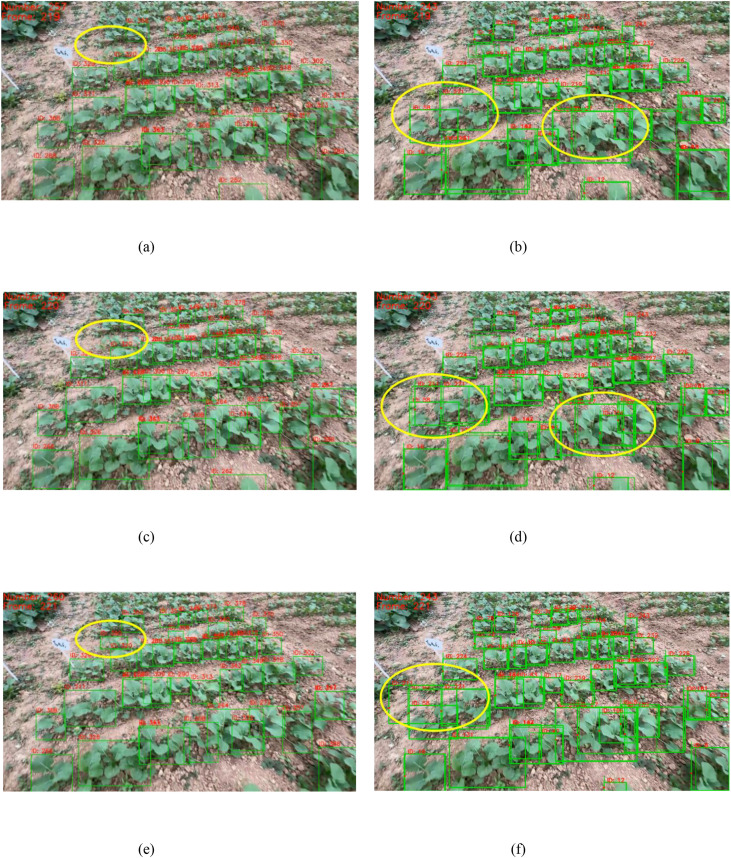
**(A, C, E)** Examples of three consecutive frames starting from the 219th frame of Video 4 using the SORT-based adaptive method, where most rapeseed seedlings maintained their IDs; **(B, D, F)** examples of three consecutive frames starting from the 219th frame of Video 4 using the DeepSORT-based adaptive method, where IDs of rapeseed seedlings in dense regions changed significantly. Yellow circles indicate regions with changed counting boxes.

**Figure 14:**
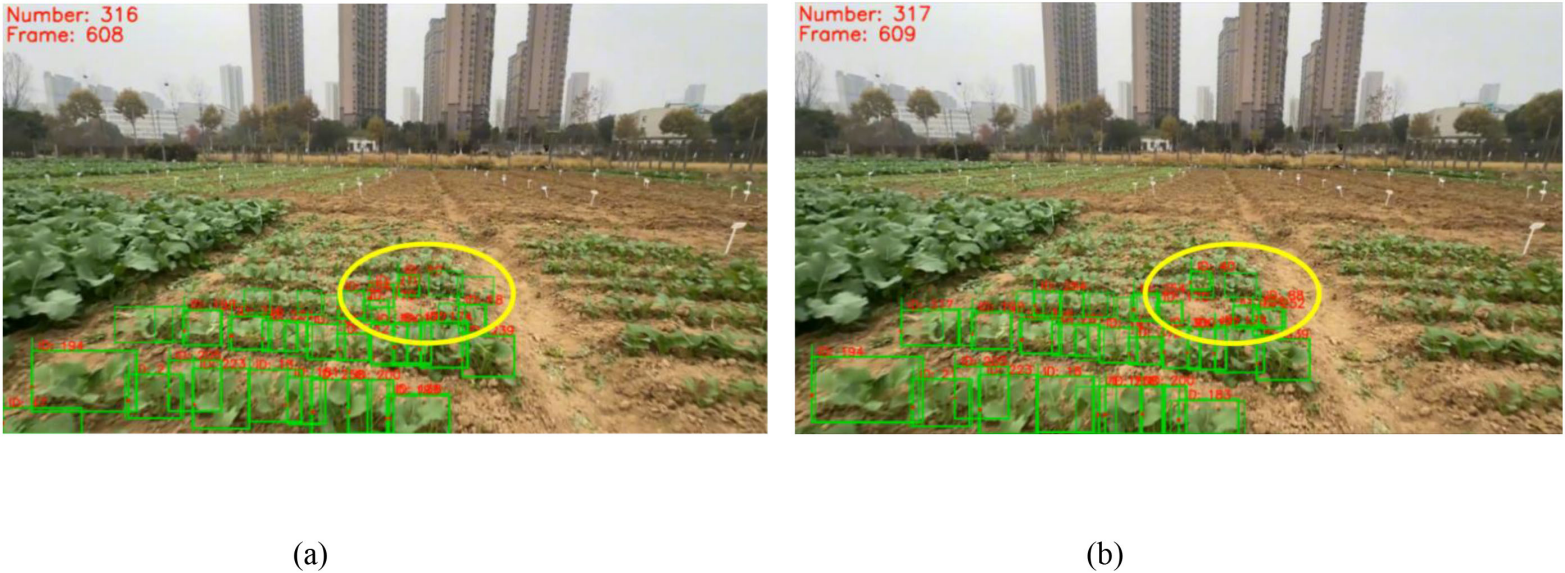
**(A, B)** shows two consecutive frames randomly selected from Video 14, showing large fluctuations in the tracking stability of DeepSORT. Yellow circles indicate regions with changed counting boxes.

The SORT tracking algorithm exhibited good tracking performance in rapeseed fields. As shown in **Figure 12**, for 20 videos using the adaptive counting method based on SORT, the average ID switch rate (*W_ID_*) was 8.47%, meaning most rapeseed seedlings (even those with severe mutual occlusion) maintained their IDs from appearance to disappearance, as shown in **Figures 13a, c, e**. In contrast, for the same videos with three consecutive frames using the adaptive counting method based on DeepSORT, IDs of rapeseed seedlings in dense regions changed significantly, as shown in **Figures 13b, d, f**. For the counting method based on SORT, the average target tracking accuracy (*P_tr_*) and average target tracking precision (*P_mt_*) were 87.53% and 89.75%, respectively. For videos using the counting method based on DeepSORT, the *W_ID_*, *P_tr_*, and *P_mt_* were 36.05%, 71.50%, and 54.72%, respectively. The *P_mt_* of the SORT-based counting method was 35.03% higher than that of DeepSORT, indicating that SORT is far superior to DeepSORT in terms of trajectory continuity and stability. Additionally, two consecutive frames randomly selected from a video using the DeepSORT tracking algorithm are shown in **Figure 14**. As can be seen from **Figures 13b, d** and **14**, DeepSORT exhibits large fluctuations in tracking stability, which possibly due to its poor robustness in dense or occluded scenarios in this study.

**Figure 15:**
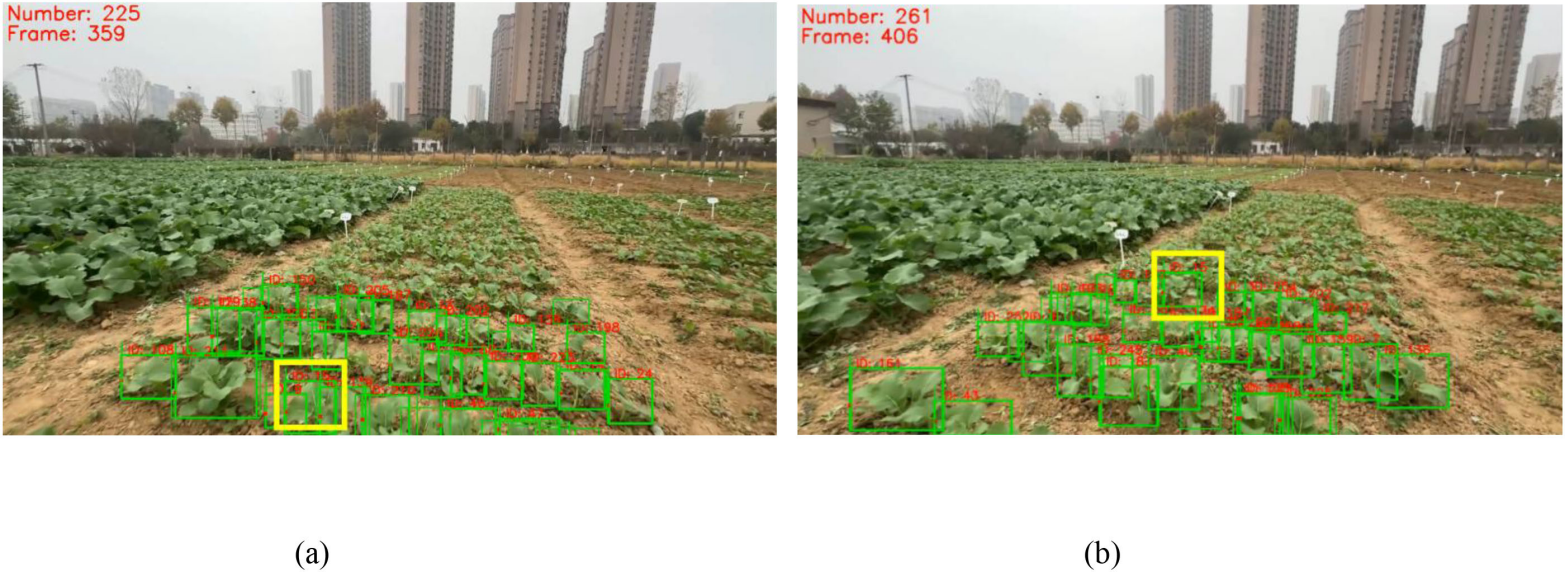
Counting results of the DeepSORT-based adaptive method, showing rapeseed seedlings with ID 15 in the 359th and 406th frames of Video 14; **(A)** shows the 359th frame, **(B)** shows the 406th frame.

Compared to the DeepSORT-based counting method, the SORT-based counting method was more accurate. As shown in **Figure 9**, counting results of the conventional and adaptive methods based on DeepSORT for Videos 1 to 10 were 0.55 and 0.79 times the *GT* value, respectively, while those for Videos 11 to 20 were only 0.4 and 0.45 times the *GT* value, far lower than the corresponding results of the SORT-based counting method. This may be because the DeepSORT-based counting method introduces a deep appearance feature extractor (Zhang et al., 2024), which struggles to learn subtle features that sufficiently distinguish highly similar plants. In the same video, IDs already assigned to rapeseed seedlings may be reassigned to new rapeseed seedlings, as shown in **Figure 15**, leading to generally lower counting results compared to the *GT* value. The highly similar appearance of rapeseed seedlings significantly affects counting accuracy, which is also the reason for the high *W_ID_* of DeepSORT. Additionally, DeepSORT relies more on static background assumptions (Huang et al., 2024), while data in this study was acquired by staff walking in the field with a gimbal, with fixed rapeseed seedlings, undermining the reliability of DeepSORT’s motion prediction and appearance matching. In the environment of this study, the tracking performance of DeepSORT was far inferior to that of SORT. Therefore, the CropTriangulator method combining the adaptive method with SORT provides considerable advantages for real-time counting of rapeseed seedlings.

The main sources of counting errors in this study include target overlap, false positives (*FP*), and false negatives (*FN*): late-stage rapeseed seedlings have dense foliage, leading to 30-40% overlap in the 90° vertical view but 15-20% in the 45° oblique view, which causes the model to misclassify multiple seedlings as a single target and results in FN, for example, in Video 18 (90° view), 12 out of 38 *FN* errors (31.6%) were attributed to severe leaf overlap, while the 45° oblique view reduces overlap by exposing more 3D structural features (e.g., stem-root separation), and the SORT algorithm’s motion prediction further mitigates this by maintaining ID continuity for partially occluded seedlings; *FP* primarily originate from non-target regions (e.g., weeds, soil clods) and perspective-induced misdetection, and the AdapDBSCAN algorithm filters 89.2% of *FP* by dynamic density clustering, but residual *FP* (accounting for 2.3% of total counts) still occur in edge regions of drainage ditches due to the pixel-to-distance ratio in edge regions deviating from the central area, leading to over-clustering; beyond overlap, *FN* are caused by motion blur (18.7% of *FN*) and small-sized seedlings in distant regions (22.1% of *FN*). Motion blur (resulting from walking speed fluctuations) reduces detection confidence, while distant seedlings (less than 5 pixels in diameter) are easily missed by YOLOv11n.

To verify the superiority of the CropTriangulator method in late-stage rapeseed seedling counting, four representative SOTA crop counting methods were selected for benchmarking. The comparison is based on publicly reported performance metrics from the original studies, with a focus on counting accuracy. The comparative results were summarized in **Table 5**, where the performance of existing methods was extracted from their original publications, and the performance of CropTriangulator was based on the 45° optimal view.

**Table 5:** Performance comparison between CropTriangulator and SOTA methods.

Reference	Method	Target Crop (Stage)	Counting Accuracy	CropTriangulator Accuracy (45° View)
Zhao et al. (2018)	UAV Image and Deep Learning Detection	Rapeseed (Early/Late)	83.67%	97.13%
Lin et al. (2022)	Improved YOLOv5s and DeepSORT	Peanut (Seedling)	98.08%	97.13%
Tan et al. (2023)	Anchor-Free Deep Convolutional Neural Network for Tracking and Counting	Cotton (Seedling/Flower)	94.40%	97.13%
Rong et al. (2023)	RGB-D Fusion and Improved YOLOv5 Detection and Multi-Object Tracking	Tomato (Cluster)	97.90%	97.13%

As shown in **Table 5**, CropTriangulator method demonstrates significant advantages in late-stage rapeseed counting. Compared to the only rapeseed-specific method (Zhao et al., 2018), CropTriangulator achieves a 13.46% higher accuracy, solving the bottlenecks of single-image data (partial target missing) and late-stage occlusion that limit existing rapeseed counting methods. Despite targeting more complex late-stage rapeseed (with severe leaf overlap and perspective distortion), the accuracy of CropTriangulator is comparable to early-stage peanut counting (Lin et al., 2022) and outperforms dense cotton seedling counting (Tan et al., 2023), verifying strong adaptability to complex growth stages. Unlike Rong et al. (2023) which relies on depth sensors and controlled greenhouse environments, CropTriangulator achieves comparable accuracy in open fields.

These results confirm that the CropTriangulator method effectively addresses the optimization gap of existing methods in late-stage rapeseed seedling counting, balancing high accuracy, adaptability to complex field scenarios, and practical applicability.

Although the problem of data density distribution in rapeseed seedling counting has been basically solved, several challenges remain: firstly, motion blur and shooting area offset not only affect the detection stage but also impact clustering and counting performance. Secondly, the CropTriangulator method is constrained by tracking results. Some studies indicate that existing algorithms have poor tracking accuracy for dense pedestrians, suggesting poor performance of current tracking methods in complex and variable backgrounds (Wang W. et al., 2022). Fortunately, improvements in tracking algorithms have shown positive effects in kiwifruit orchard counting (Zhang et al., 2025). However, direct cross-study comparisons are challenging due to differences in data acquisition standards. Stable relative motion during data acquisition ensures effective cross-frame matching. It should be noted that rapeseed seedlings have similar phenotypic characteristics, especially those in the same growth stage, making it difficult for existing tracking technologies with feature recognition modules to distinguish them, leading to ID assignment errors. Some studies have achieved accurate crop identification by acquiring images of crops at different growth stages in the field (Li et al., 2025; Naseer et al., 2025).

Challenges in crop counting in complex field environments require comprehensive research in the future. The adaptive dynamic parameter adjustment mechanism of AdapDBSCAN must be optimized to improve clustering performance, especially in uneven density scenarios. User-friendly video acquisition methods should also be explored to better control video quality, reduce the impact of motion errors on clustering, and thus weaken their impact on counting. Meanwhile, developing stable and efficient rapeseed seedling tracking technologies in complex agricultural scenarios remains a topic worthy of further exploration. Additionally, future research is advised to focus on temporal analysis (lv et al., 2025; Wang et al., 2024), tracking morphological changes of the same plant at different seedling stages (early and late seedling stages), which may help improve the stability of tracking methods and avoid duplicate counting.

The video-based rapeseed seedling counting method proposed in this study is more convenient than previous image-based algorithms and more easily applicable to actual crop planting. These results verify the excellent performance of the CropTriangulator method and the feasibility of eliminating rapeseed seedlings in non-target regions based on target density distribution. Therefore, this study contributes to formulating strategies to improve crop emergence rates and productivity estimation.

## 4 Conclusions

This study proposed CropTriangulator, an automatic rapeseed seedling counting pipeline that achieves accurate row-based counting with smartphone-captured videos. YOLOv11n demonstrated excellent performance in detecting seedlings against complex backgrounds while maintaining fast inference times, indicating its suitability for small-scale object detection in agricultural settings. The AdapDBSCAN method achieved promising results in row-based seedling counting by dynamically adjusting clustering parameters to filter out non-target seedlings, suggesting that adaptive density-based clustering effectively addresses perspective-induced distortions. The 45° oblique view proved significantly superior to the 90° vertical view, improving counting accuracy by effectively reducing leaf occlusion and providing more discriminative phenotypic features of seedlings.

Although the pipeline showed reliable row-based counting, results were affected by the inability to preview frames during data collection. The study did not consider the impact of path offsets or camera shakes, which may limit the applicability of this method in different field conditions. Future research should focus on developing more efficient approaches to achieve row-based rapeseed seedling counting based on video, along with user-friendly video capture techniques and more precise shooting views. Furthermore, it would be beneficial to integrate temporal growth analysis to improve tracking consistency across seedling stages. With further optimization, this pipeline holds significant potential for automated yield estimation, supporting data-driven field management decisions.

## Data Availability

The raw data supporting the conclusions of this article will be made available by the authors, without undue reservation.
